# Economic impact of clinical pharmacist interventions in a general tertiary hospital in Qatar

**DOI:** 10.1371/journal.pone.0286419

**Published:** 2023-06-01

**Authors:** Dina Abushanab, Mounir Atchan, Reem Elajez, Mohamed Elshafei, Ahmed Abdelbari, Moza Al Hail, Palli Valapila Abdulrouf, Wessam El-Kassem, Zanfina Ademi, Abdalla Fadul, Elmustafa Abdalla, Mohammad Issam Diab, Daoud Al-Badriyeh

**Affiliations:** 1 Department of Pharmacy, Hamad Bin Khalifa Medical City, Hamad Medical Corporation, Doha, Qatar; 2 Department of Pharmacy, Hamad General Hospital, Hamad Medical Corporation, Doha, Qatar; 3 Centre for Medicine Use and Safety, Faculty of Pharmacy and Pharmaceutical Sciences, Monash University, Melbourne, Australia; 4 School of Public Health and Preventive Medicine, Monash University, Melbourne, Australia; 5 Department of Medicine, Hamad Medical Corporation, Doha, Qatar; 6 College of Pharmacy, QU Health, Qatar University, Doha, Qatar; University of Turin, ITALY

## Abstract

**Background:**

With an increasingly strained health system budgets, healthcare services need to continually demonstrate evidence of economic benefits. This study sought to evaluate the economic impact of interventions initiated by clinical pharmacists in an adult general tertiary hospital.

**Methods:**

A retrospective review of clinical pharmacist interventions was carried out throughout follow-up durations in March 2018, July/August 2018, and January 2019 in Hamad General Hospital (HGH) at Hamad Medical Corporation (HMC) in Qatar. The study included clinical pharmacy interventions data of patients admitted to the internal medicine, critical care, and emergency wards. Included interventions were documented by clinical pharmacists or clinical pharmacy specialists, and approved by physicians. Interventions by non-clinical pharmacists or with missing data were excluded. Adopting the perspective of HMC, we calculated the total economic benefit, which is the sum of the cost savings and the cost avoidance associated with the interventions. Cost savings was defined as the reduced cost of therapy associated with therapy changes minus the cost of intervention and cost avoidance was the cost avoided by eliminating the occurrence of adverse drug events (ADEs). Sensitivity analyses were performed to assess the robustness of results against uncertainties.

**Results:**

A total of 852 interventions, based on 340 patients, were included. The analysis projected an annual total benefit of QAR 2,267,036 (USD 621,106) based on a negative cost-savings of QAR-175,139 (USD-47,983) and a positive cost avoidance of QAR741,898 (USD203,260) over the 3-month follow-up period. The uncertainty analysis demonstrated the robustness of outcomes, including a 100% probability of positive economic benefit.

**Conclusions:**

The clinical pharmacist intervention was associated with an increased cost of resource use, which was overtaken by the cost avoidance generated. The pharmacy intervention, therefore, is an overall economically beneficial practice in HGH, reducing ADEs with considerable consequential positive economic savings.

## Introduction

The irrational use of medicine remains a serious public health problem worldwide [1]. The World Health Organization estimates that more than half of all medications are prescribed or dispensed inappropriately, which may cause adverse outcomes such as medication-related problems (MRPs), increased hospitalisation due to adverse drug events (ADEs), and increased healthcare costs [2]. In the United States (US), a round 5% of ADEs occur in hospitalized patients [3]. A report from France also showed that 40% of ADEs, which occurred during hospitalisation, comprised medications, where half of these were preventable [4]. Additionally, a large population study in the US showed that mortality rates due to ADEs were 19.18% higher with 1,971 excess death than those without ADEs [5].

ADEs not only impose a huge health burden but also increase expenditures on medications by almost 70% [6] and overall healthcare costs by USD 11,486 per ADE [7]. In the context of demonstrated ADEs prevention and reduced healthcare costs, clinical pharmacists have become an integral part of the multidisciplinary management team and play a key role within the healthcare system [8]. During their routine practice, clinical pharmacists perform clinical interventions, defined as “any action taken by a pharmacist that directly results in a change of patient management or therapy” [9]. Strong evidence now exists to support the importance of clinical pharmacist interventions that are designed to reduce the ordering of inappropriate medication regimens [10]. The extent of the potential to offset total healthcare costs varies [9, 11–14], however, as per study settings and methods used. Here, understanding the economic impact of local clinical pharmacy interventions on resource use is important for any setting, including assisting decision and policymakers in better justifying the support of the clinical pharmacy services with salaries for personnel, for example. In this study, we sought to analyze the economic impact of clinical pharmacists’ interventions against ADEs in the largest adult general tertiary setting in Qatar.

## Materials and methods

### Study design and setting

A retrospective analysis of clinical pharmacy interventions was carried out in Hamad General Hospital (HGH), a general tertiary hospital with 603 beds serving adult patients, including internal medicine, emergency medicine, surgery, and critical care at Hamad Medical Corporation (HMC) in Qatar [15].

At the time of the study, there were around 15 clinical pharmacists employed in the internal medicine, emergency department, and adult intensive care unit in HGH. The role of clinical pharmacy practice in Qatar has advanced in recent years, with clinical pharmacists providing a range of direct patient care and cognitive services, particularly in secondary care. They participate in clinical rounds and discussions and contribute to patient history review including diseases and medications, identification of ADEs, therapeutic recommendations, individualization of dosage regimen, medication reconciliation, patient education, and patient counselling. They are also involved in leading the anticoagulation clinic and provision of health information about the use of medical devices such as inhalers and insulin pens. In addition to this, a clinical pharmacist can also promote cost-effective medication recommendations.

All clinical interventions were directly obtained via the clinical intervention sheet, which is included in each patient record in the Cerner electronic medical database. Incomplete information in the clinical interventions intervention sheet was retrieved and extracted from the patient’s file notes in the electronic medical records. The patient medical records have all the details of patient management, including the interventions documented in the clinical pharmacy interventions sheets. Analysed interventions in this study were documented by clinical pharmacists or clinical pharmacy specialists, and approved by physicians.

### Ethics approval

This study was approved by the Medical Research Center (MRC) of Hamad Medical Corporation (HMC), Qatar (MRC-01-19-110) on the 18^th^ September 2019 (S1 File). Given the retrospective nature of the study, based on medical records, the MRC ethics committee waived the requirement for informed consent.

### Clinical interventions

Clinical pharmacist interventions were defined as any action by a pharmacist that directly resulted in a change to patient management or therapy [16]. The clinical pharmacy interventions in HMC are provided as part of the routine working-day tasks performed by clinical pharmacists. Staff/operational (non-clinical) pharmacists may suggest interventions, but these have to ideally be documented through communication with clinical pharmacists looking after patients. The patients’ pharmacotherapeutic follow-up is performed through a daily review of patients’ medical records by the clinical pharmacists, including medications and laboratory tests, where the need for intervention can be identified.

### Study population

In this study, clinical pharmacy interventions data were for adult patients admitted to the internal medicine, critical care, and emergency wards at HGH. The study sample took place over a period of non-successive 3 months: in March 2018, from July 15 to August 15, 2018, and in January 2019. In HMC, the annual performance evaluation takes place between January and February, and it is possible that the documentation of interventions by clinical pharmacists could be influenced by this annual performance evaluation, whereby they are possibly more vigilant. Therefore, the data collection of interventions was based on the first month after the annual staff performance evaluation, the last month of the year before the annual performance evaluation, and the middle month of the year.

#### Inclusion criteria

- Interventions were for patients for whom at least one medication was indicated for continued use during hospitalisation.
- The interventional recommendations that were accepted by the physicians and were, therefore, implemented.

#### Exclusion criteria

- Patient medical records with missing data, about interventions that details that could not be initially extracted from the clinical pharmacy intervention sheets, were excluded from the study.
- Interventions were directly performed by a staff/operational pharmacist (non-clinical), without going through the clinical pharmacist looking after the patient.
- Interventions that were rejected by the clinicians.

All eligible patients were followed-up from the time of admission to discharge.

### Economic analysis

Cost savings, defined as the reduced cost of therapy because of the intervention, was calculated by subtracting the cost of after-clinical pharmacy intervention therapy from the cost of before-clinical pharmacist intervention therapy when this is in positive values. The cost of after intervention was based on the actual original therapy duration until intervention, added to the cost of the alternative therapy (therapy after the change) based on the duration of its full course. The cost before intervention was based on the duration of therapy before intervention. In this analysis, a 3-month prescription refills cost was considered for chronic disease medications, while for acute diseases, we considered the duration as per the prescription order or the national HMC guideline.

The following is an example of a cost saving calculation scenario:

- A patient with reduced creatinine clearance (i.e. 14 mL/minute) was prescribed piperacillin and tazobactam 2.25 g intravenous every 6 hours. The clinical pharmacist, however, recommended dose adjustment of piperacillin and tazobactam to be 2.25 g intravenous every 8 hours.
- The total cost before intervention was calculated to be QAR 610 (USD 167), whereas the total cost after intervention was calculated to be QAR 457 (USD 125). Hence, to generate the reduced cost associated with the intervention, QAR 457 (USD 125) was subtracted from QAR 610 (USD 167). This yields QAR -153 (USD 37).

Cost avoidance, defined as eliminating a potential increase in the costs related to ADEs, was calculated for each intervention by multiplying the estimated probability of an ADE in the absence of the intervention by the cost of an ADE using the Nesbit et al. method [17]. The Nesbit et al. method assigns a probability score to each clinical intervention based on the severity of ADE. The probabilities are: 0 (none), 0.01 (very low), 0.1 (low), 0.4 (medium), or 0.6 (high) [17]. In line with other studies, it was assumed that an ADE will lead to an increase in hospitalisation by two days [18, 19]. The value of the daily hospital stay was based on the ward that the patient was in. Then, the total cost avoidance was calculated for all interventions for MRPs. Table 1 provides a description about Nesbit et al. method [17].

**Table 1:** Patients’ demographics among the study periods.

	Total	March 2018 (n = 102)	July-August 2018 (n = 86)	January 2019 (n = 152)	P value
Variable	Average ± standard deviation or frequency (%)				
Gender					
Male	222 (65.29)	85 (83.33)	14 (16.28)	123 (80.92)	0.13
Female	118 (34.71)	17 (16.67)	72 (83.72)	29 (19.08)	
Age	51.04 ± 17.66	48.33± 18.12	50.23 ± 18.03	53.08 ± 16.96	0.16
Weight	77.49 ± 26.63	76.67 ± 28.18	74.29 ± 23.72	78.67 ± 20.65	0.23
Nationality					
Arab	188 (55.29)	52 (50.98)	45 (52.33)	91 (59.87)	0.11
Asian (non-Arab)	117 (34.41)	46 (45.10)	15 (17.44)	56 (36.84)	
African (non-Arab)	30 (8.82)	3 (2.94)	25 (29.07)	2 (1.32)	
Others	5 (1.47)	1 (0.98)	1 (1.16)	3 (1.97)	
Ward type					
Internal medicine	183 (53.82)	45 (44.12)	52 (60.47)	86 (56.58)	0.09
Emergency	105 (30.88)	38 (37.25)	21 (24.42)	46 (30.26)	
Critical care	52 (15.29)	19 (18.63)	13 (15.12)	20 (13.16)	

The key economic outcome of our study is the total economic benefit, defined as the sum of the cost savings and the cost avoidance, minus the cost associated with the intervention. All costs were calculated as per the 3-month study period. An annual projection of the total economic benefit was also calculated by multiplying the 3-monthly overall benefit by 4.0.

### Panel members

In accordance with the Nesbit et al. method [17], a panel of experts was used to identify the probabilities of ADEs in the absence of interventions [17]. The panel members were of six healthcare professionals; four clinical pharmacists with over eight years of clinical experience and two resident physicians specialized in internal medicine, critical care, and infectious disease at HGH. For each included clinical intervention, each clinical pharmacist provided an estimate of the likelihood of an ADE in the absence of the intervention, and an average probability estimate was calculated between members. The two physicians validated the generated probability estimates of ADEs. Disagreements were further discussed among members until consensus.

### Resource inputs

The analysis included medication and non-medication resources (including laboratory, diagnostic, and hospital stay), with the cost of which extracted from the pharmacy and finance departments at HMC. All costs were based on the financial year 2022, utilizing the Qatari health Consumer Price Index [20], and were presented in Qatari Riyal (QAR) and the United States Dollar (USD); 1 USD = 3.65 QAR.

### Perspective

The economic analysis was performed from the HGH hospital perspective, accounting for direct medical costs only. Other types of cost, such as indirect and non-medical costs, were not considered.

### Sample size

This study was not comparative, and there are no standardization or relevant sample size calculations for the purpose of this analysis. In any case, unlike clinical research, the current economic evaluation is not concerned in terms of hypothesis testing, but it is about making cost estimations. An underpowered economic evaluation still provides important information that guides decision making [21, 22]. A total period of non-successive 3 months is believed to provide a representative sample of the clinical pharmacy interventions in a year.

### Statistical analysis

Numerical and percentage measures were used for categorical variables, while mean and standard deviation measures were used for continuous variables. Because of the non-successive nature of the three follow-up months in this study, we needed to confirm the homogeneity of treated patients and that there are no considerable demographic shifts that may affect practices, including to increase certainty about the projected annual economic benefit. Hence, a one-way analysis of variance (ANOVA), Kruskal–Wallis tests, and Chi-Square tests were used to determine the significant difference among the three groups: (i) March, 2018, (ii) July 15 to August 15, 2018, and (iii) January, 2019. All statistical analyses were performed using the IBM SPSS (Statistical Package for the Social Sciences) version-24.

### Sensitivity analysis

Sensitivity analyses were undertaken to explore the uncertainty surrounding main cost and probability inputs. A deterministic sensitivity analysis (DSA), targeting one uncertain input variable at a time, was performed to assign a ±20% variation range of the base case value of the cost of the ADE, using a triangular type of random value distribution. A probabilistic sensitivity analysis (PSA), targeting several probabilistic inputs at once, was used to assign an uncertainty range of ±15% of the base case values of the probabilities of ADEs, using a triangular-type distribution, and based on 10,000 simulations. All analyses were performed via Monte Carlo simulation, using @Risk-5.7 (Palisade Corporation, NY). All outcomes were presented graphically.

## Results

### Description of patients and interventions distribution

During the study period, a total of 852 interventions for 340 patients were identified by the clinical pharmacists. Of these, 252 interventions were performed in 102 patients in March 2018, 262 interventions occurred in 86 patients in July-August 2018, and 338 interventions occurred in 152 patients in January 2019. The mean patient age was 51.04 ± 17.66 years, and the sex ratio (male/female) was nearly 2:1. Most of the patients were hospitalised in the internal medicine ward, followed by emergency units, and most of the patients were as expected Arabs, followed by Asians. No statistically significant differences were detected between the study follow-up groups. Table 1 presents the patients’ demographics among the study period groups.

### Types of the interventions

A total of 453 (53.17%) interventions took place in the internal medicine ward, 253 (29.69%), in the critical care, and 146 (17.14%) in the emergency department. Our analysis showed that the most common interventions intercepted by the clinical pharmacists were related to appropriateness of therapy, i.e. 541 (63.50%) interventions, followed by interventions related to dosing and administration, i.e. 287 (33.69%). Only 13 (1.53%) interventions were related to contraindications, 6 (0.7%) were related to drug interaction, and those related to duplicate therapy necessitating discontinuation were 5 (0.6%). We also found that the most prevalent interventions were related to anti-infective and cardiovascular medications. In Table 2, we present a description of the categories of the interventions with examples, and the associated average probability of avoided ADEs as per category.

**Table 2:** Examples of clinical pharmacy interventions and description of the probability of avoided adverse drug events.

Averaged probability of avoided ADE	Probability of ADE category	Categories of intervention resources with examples
0.01	Very low	Discontinuation of a medication; change in dosage form; and change in medication strength.
		***Example*:** Patient admitted to the internal medicine ward with nausea and vomiting due to viral gastroenteritis and was given ondansetron 4 mg oral twice daily. The patient improved and was recommended to switch to ondansetron 4 mg oral as needed.
0.1	Low	Discontinuation of a medication; addition of another medication; switching to alternative medication; increase in medication duration; change in dosage form; increase in medication frequency; change in medication strength; increase in medication dose; decrease in medication duration; decrease in medication frequency; decrease in medication dose; requesting a lab test; requesting a diagnostic test; requesting a TDM; addition of a prophylactic agent during hospitalisation.
		***Example*:** Patient with hypotension (blood pressure 84/58 mmHg) due to unknown reason, was admitted to the emergency department. The patient received 2 mg/min norepinephrine which resulted in improvement in blood pressure (i.e. blood pressure 110/86 mmHg). However, the prescription was still active, therefore the pharmacist recommended to discontinue medication.
0.2	Low to medium	Discontinuation of a medication; addition of another medication‘ switching to alternative medication; increase in medication duration; change in dosage form; increase in medication frequency; change in medication strength; increase in medication dose; decrease in medication duration; decrease in medication frequency; decrease in medication dose; requesting a lab test; requesting a diagnostic test; requesting a TDM; and addition of a prophylactic agent during hospitalisation.
		***Example*:** Patient with seasonal influenza and normal renal function on oseltamivir 30 mg orally twice daily. The pharmacist recommended to increase the dose to 75 mg twice daily for 5 days because 30 mg twice daily is used for renally impaired patient.
0.3	Low to medium	Discontinuation of a medication; addition of another medication; switching to alternative medication; addition of a prophylactic agent during hospitalisation, increase in medication dose; increase in medication frequency; increase in medication duration; addition of a prophylactic agent increase during hospitalisation; requesting a lab test; requesting a TDM; decrease in medication dose; decrease in medication frequency; decrease in medication duration; requesting a diagnostic test; change in dosage form; change in medication strength.
		***Example*:** Patient with hypertension on lisinopril 20 mg/hydrochlorothiazide 12.5 mg once daily, was admitted due to high blood pressure (139/87 mmHg). The potassium level was 3.3 mmol/L and was recommended to initiate potassium chloride 20 mEq orally three time daily.
0.4	Medium	Discontinuation of a medication; addition of another medication; switching to alternative medication; increase in medication dose; addition of a prophylactic agent during hospitalization; requesting a lab test, TDM; increase in medication duration; decrease in medication dose; requesting a diagnostic test.
		***Example*:** Critically ill patient with infection extended spectrum beta-lactamase infection detected in urine. The patient was empirically initiated on piperacillin-tazobactam 2.25 g intravenously every 8 hours. However, the pharmacist recommended to discontinue piperacillin-tazobactam and initiate ertapenem 500 mg intravenous once daily as culture showed sensitivity to ertapenem.
0.5	Medium to high	Discontinuation of a medication; addition of another medication; switching to alternative medication; increase in medication dose; decrease in medication dose; addition of a prophylactic agent during hospitalisation; requesting a lab test; increase in medication duration.
		***Example*:** Critically ill patient with suspected sepsis, was on piperacillin-tazobactam 2.25 g intravenous every 6 hour which is lower than the therapeutic dose for normal renal function. The pharmacist recommended to increase the dose to 4.5 g intravenous every 6 hour.
0.6	High	Discontinuation of a medication; addition of another medication; switching to alternative medication.
		***Example*:** Patient was on terlipressin 2 mg intravenously every 6 hours to manage oesophageal varices bleeding. The pharmacist recommended to discontinue terlipressin because patient developed electrocardiograph changes. No alternative was given.

*ADE: adverse drug event

### Cost analysis

#### Cost saving

It is estimated that the overall added cost associated with interventions over a 3-month period was found to be approximately QAR 224,551 (USD 61,521) and the overall reduced cost due to the interventions was QAR 49,412 (USD 13,538). The overall cost savings due to the pharmacy lead interventions was therefore in negative, i.e. QAR -175,139 (USD -47,983). Of which, change in medication dose (i.e. incorrect dose, increased dose or decreased dose) and switching to alternative medication contributed to the cost the most, while the change in medication strength and discontinuation of a medication contributed to the cost the least. In terms of added cost, the addition of a medication had the highest impact, while the change in medication strength had the lowest impact. The added and reduced costs with each category of intervention are available in Table 3.

**Table 3:** Cost saving, added cost, and cost avoidance associated with each category of clinical pharmacist intervention during the study period.

Type of interventions	Overall reduced cost associated with interventions, QAR (USD)	Overall added cost associated with interventions, QAR (USD)	Overall cost avoidance, QAR (USD)
Addition of another medication (n = 233)	0	134,249 (36,781)	262,938 (72,038)
Discontinuation of a medication (n = 174)	671 (184)	0	133,542 (36,587)
Addition of a prophylactic agent during hospitalisation (n = 35)	0	35,635 (9,763)	44,514 (12,196)
Switching to alternative medication (n = 41)	11,669 (3,197)	4,165 (1,141)	37,095 (10,163)
Change in medication dosage form (n = 51)	9,526 (2,610)	2,870 (786)	27,450 (7,521)
Change in medication strength (n = 41)	416 (114)	81 (22)	29,676 (8,130)
Therapeutic drug monitoring (n = 31)	0	940 (258)	22,257 (6,098)
Change in medication dose (i.e. incorrect dose, increase dose or decrease dose) (n = 132)	23,764 (6,511)	40,061 (10,976)	126,123 (34,554)
Change in medication frequency (n = 38)	1,291 (354)	1,962 (538)	23,741 (6,504)
Change in medication duration (i.e. incorrect duration, increase duration or decrease duration) (n = 38)	2,075 (568)	1,101 (302)	5,935 (1,626)
Addition of a diagnostic test (n = 7)	0	2,109 (579)	5,700 (1,562)
Addition of a lab test (n = 29)	0	1,258 (345)	21,515 (5,895)
Addition of a culture test (n = 2)	0	120 (33)	1,412 (387)
Addition of a vaccine	0	0	0
Total	49,412 (13,538)	224,551 (61,521)	741,898 (203,260)

*QAR: Qatari Riyal, USD: United States Dollar (1 USD = 3.65 QAR)

#### Cost avoidance

The probability of ADEs in the absence of interventions with an average of 0.01 was calculated for each of 8 interventions; 0.1 for 100; 0.2 for 242; 0.3 for 310; 0.4 for 116; 0.5 for 69; and an average of 0.6 for 7 interventions. The overall cost avoidance due to the interventions over a 3-month period was QAR 741,898 (USD 203,260). Table 3 summarizes the cost avoidance associated with each category of intervention.

#### Total benefit analysis

Over the 3-month period of follow up, the total benefit (i.e. sum of cost saving and cost avoidance) was QAR 566,759 (USD 155,276). The average total benefit per patient or per intervention was QAR 1,667 (USD 457) or QAR 665 (USD 182), respectively. The projected total benefit per 1 year was QAR 2,267,036 (USD 621,106).

### Sensitivity analysis

**Fig 1:**
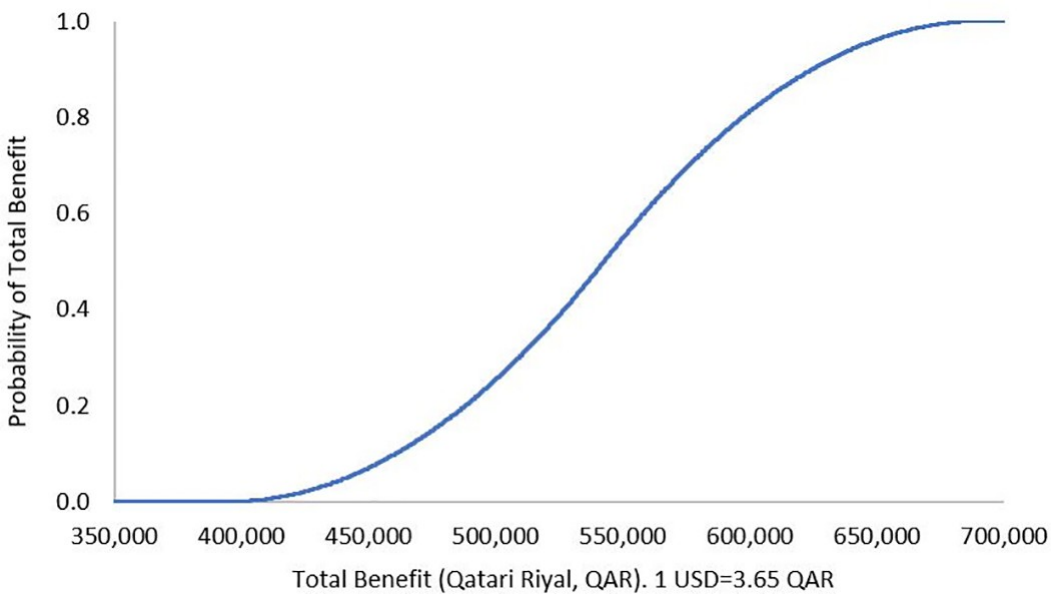
Total benefit probability curve over 3-month period (deterministic sensitivity analysis).

**Fig 2:**
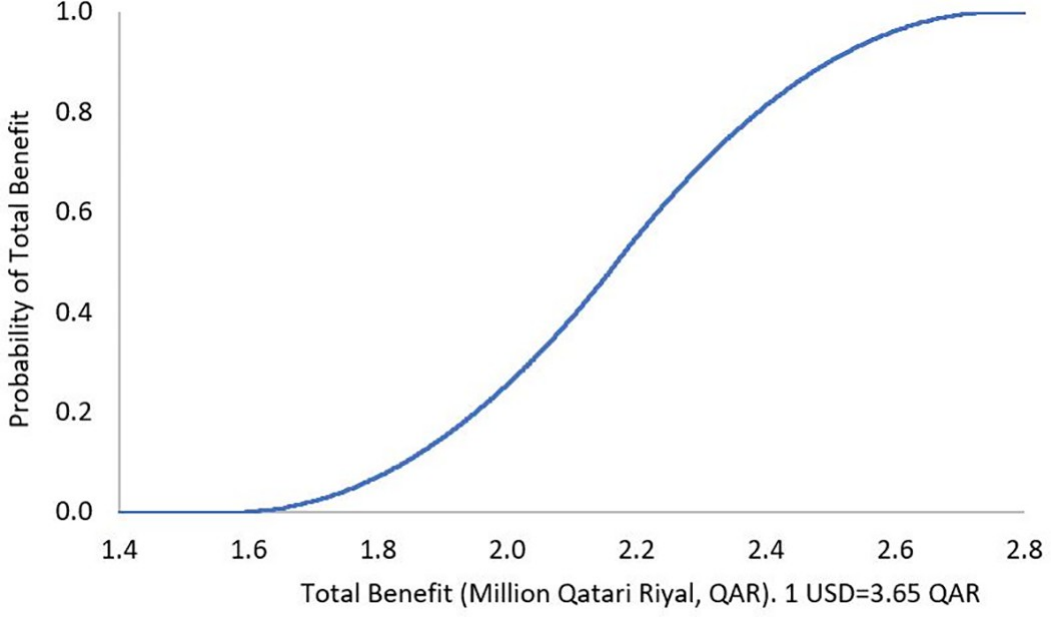
Total benefit probability curve over 1-year period (deterministic sensitivity analysis).

**Fig 3:**
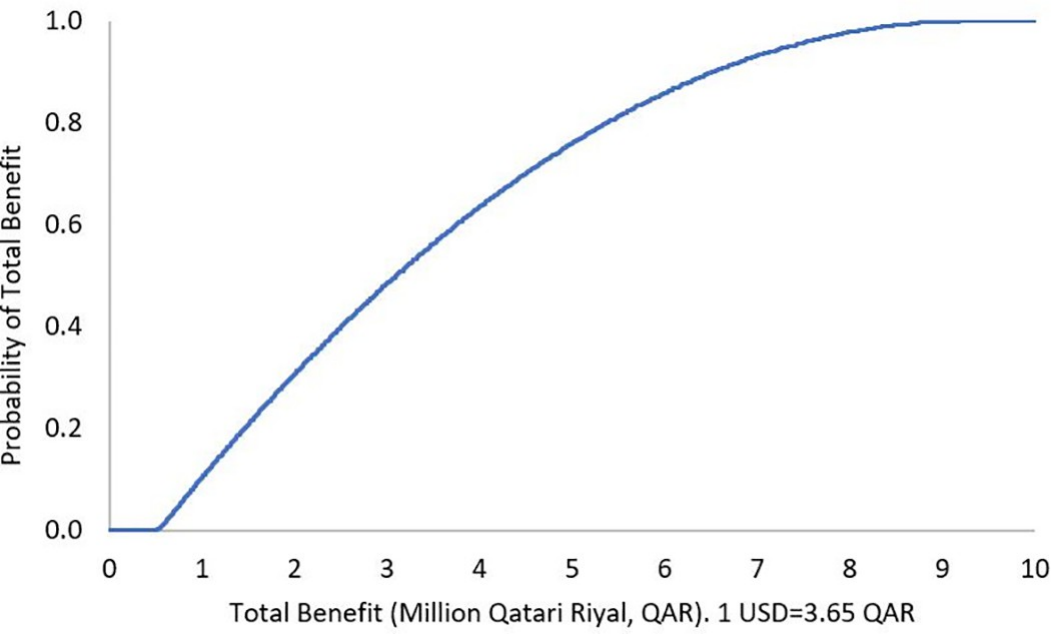
Total benefit probability curve over 3-month period (probabilistic sensitivity analysis).

**Fig 4:**
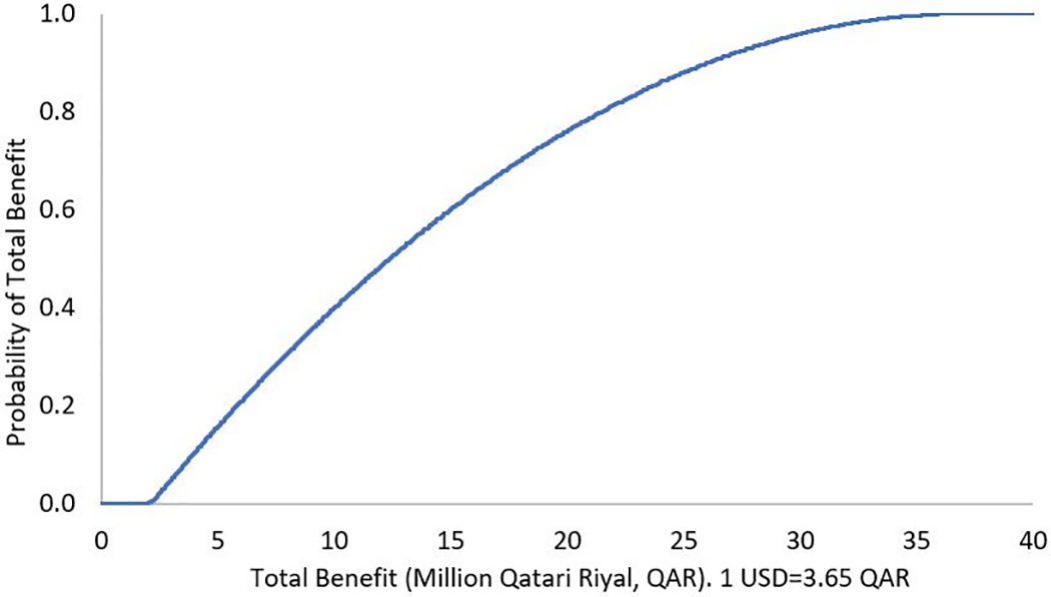
Total benefit probability curve over 1-year period (probabilistic sensitivity analysis).

The results of DSA demonstrated robustness against the uncertainty in the cost of the ADE, where the mean of total benefit over a 3-month analysis period was QAR 542,482 (USD 148,625), 95% confidence interval (CI) QAR 400,201 to 686,660 (USD 109,644 to 188,126), and the mean of total benefit over 1-year was QAR 2,169,928 (USD 594,501), 95% CI QAR 1,596,185 to 2,741,299 (USD 437,310 to 751,041) (Figs 1 and 2). The PSA showed that there is a 100% probability that the pharmacist lead intervention is associated with positive total benefit over a 3-month analysis period, with a mean of QAR 3,450,361 (USD 945,304), 95% CI QAR 487,175 to 9,182,433 (USD 133,473 to 2,515,735), as well as positive annual total benefit, with a mean of QAR 13,800,404 (USD 3,780,931), 95% CI QAR 2,016,665 to 36,506,180 (USD 552,511 to 10,001,693), Figs 3 and 4. To note, Figs 1–4 demonstrate the probability (y axis) of each of the potential generated total economic benefit values (x axis), based on the 1,000 model iterations.

**Fig 5:**
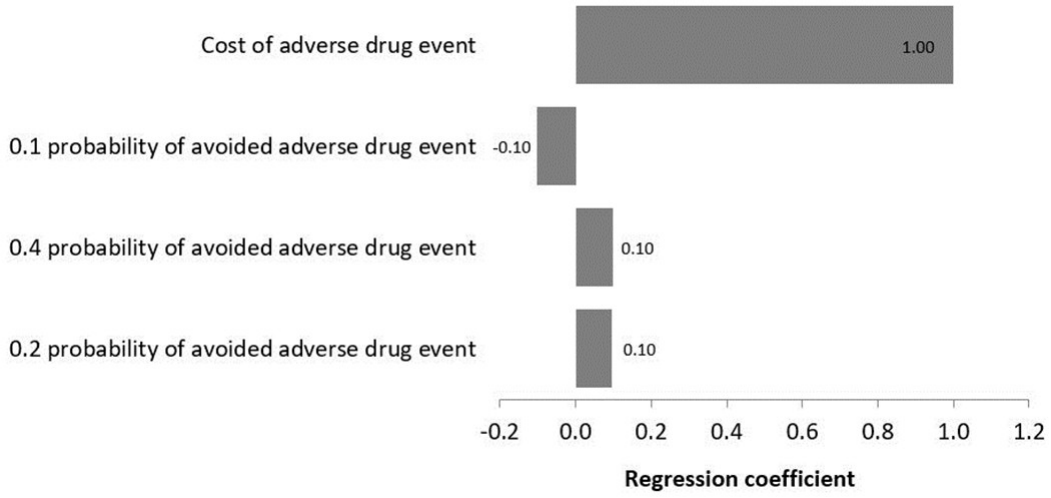
A regression tornado diagram of elements and their effect on the outcome.

A regression Tornado analysis demonstrated that the main contributor to the outcome was the cost of ADE, followed by 0.1 probability of avoided ADE, while 0.2 probability of avoided ADE was the least contributor (Fig 5). Table 4 shows the results of sensitivity analyses with their uncertainty distributions.

**Table 4:** Outcomes of sensitivity analysis with their uncertainty distributions.

Variable	Point estimate, QAR (USD)	Variation range	Projected total benefit per 1-year range, QAR (USD)	Total benefit per 3-month, QAR (USD)
One-way sensitivity analysis				
Cost of adverse drug event	1,660 (456)	Triangular distribution, QAR 1,328, 1,660, 1,992 (USD 365, 456, 547)	Mean: 2,169,928 (594,501), 95% CI 1,596,185 to 2,741,299 (437,310 to 751,041)	Mean: 542,482 (148,625), 95% CI 400,201 to 686,660 (109,644 to 188,126)
Multivariate uncertainty analysis				
Very low probability for ADE	0.01	Triangular distribution, 0.009,0.01,0.012	Mean: 13,800,404 (3,780,931), 95% CI 2,016,665 to 36,506,180 (552,511 to 10,001,693)	Mean: 3,450,361 (945,304), 95% CI 487,175 to 9,182,433 (133,473 to 2,515,735)
Low probability for ADE	0.1	Triangular distribution, 0.09,0.1,0.12		
Low to moderate probability for ADE	0.2	Triangular distribution, 0.17,0.2,0.23		
Low to moderate probability for ADE	0.3	Triangular distribution, 0.26,0.3,0.35		
Moderate probability for ADE	0.4	Triangular distribution, 0.34,0.4,0.46		
Moderate to high probability for ADE	0.5	Triangular distribution, 0.43,0.5,0.58		
High probability for ADE	0.6	Triangular distribution, 0.51,0.6,0.69		

*QAR: Qatari Riyal, USD: United States Dollar, CI: confidence interval

## Discussion

Studies have shown that medicine use errors primarily occur during the process of medication prescription, dispensing and administration [11, 23]. These could be prevented through the implementation of clinical pharmacy services [24, 25] and the participation of clinical pharmacists in reviewing prescription orders during clinical rounds. This is the first study to reveal the economic impact of the clinical pharmacist interventions associated with the prevention of ADEs in an adult general tertiary hospital in Qatar. It is noteworthy that the majority of patients were Arab, which is most likely due to the fact that Arab makes up more than 50% of the total population in Qatar [26]. Add to this, male and female distributions were not equal. This, however, could be considered representative of Qatar’s population as the recent demographical statistics in the country showed that over 75% of the population consisted of males [26].

Our findings suggest that over a 3-month period, 852 clinical pharmacist interventions took place, which were mostly related to the appropriateness of therapy and dosing, where pharmacists generally are the experts. In addition, finding suggest that interventions were more in wards that accommodates larger number of patients, whereby over 50% of the interventions occurred in hospitalised patients admitted to the internal medicine ward, which is the busiest and largest ward in HGH. The interventions were important, translated into an annual projected total benefit of QAR 2,267,036 (USD 621,106). The sensitivity analyses confirmed the robustness of outcomes and revealed that the cost of ADE and, then, the 0.1 and 0.4 probabilities of avoided ADEs contributed the most to the economic outcome. This is anticipated as the cost of ADE (i.e. assumed two hospital stay days) is a main driver behind the cost avoidance, which needed to overtake the negative cost savings if the overall economic benefit of interventions is to remain positive. Here, the cost avoidance is the product of multiplying the avoided ADE cost by the probability of avoided ADE; whereby, as already indicated above, the majority of interventions were associated with probabilities of avoided ADEs that are less than 0.4, relative to higher 0.6 and 0.7 probabilities. Hence, the cost of avoided ADE is believed to be more contributing to cost avoidance that the probability of avoided ADE.

One previous study has suggested that clinical pharmacists reduce the rates of medication errors by 66% [27], and that pharmacy interventions prevent MRPs related to effectiveness and monitoring of medications by nearly 30–40% [28]. To date, there are studies that have evaluated the economic impact of clinical pharmacist interventions in specialized units such as nephrology and critical care [9, 11–14]. However, studies that evaluated the economic impact of patients admitted to a tertiary general setting regardless of the specialized unit are currently lacking. Therefore, our study findings may not be practical to compare with previous studies conducted in different settings, given the differences among clinical practices and healthcare systems across the globe. For example, Cazarim et al. [29], in a prospective cohort study performed at a neurology unit with 506 interventions, reported that the total added cost of interventions resulted in an annual average of USD 1,158, and the annual cost avoided was USD 25,536. These values are lower than those in our findings, which is expected given the fact that the analysis was limited to interventions that occurred in the neurology department, and this might underestimate the overall economic influence of clinical pharmacists. However, similar to our findings, the addition and discontinuation of medications, and change in doses (i.e. incorrect dose, increased dose or decreased dose) were the top categories of interventions associated with the cost saving [29]. Another retrospective study, by Chen et al. [19], was conducted in a nephrology ward and found that deployment of clinical pharmacists’ interventions yielded cost savings of USD 144,138, and cost avoidance of ADEs of USD 7,342,200, which are higher than in our findings. However, here again, due to the specific nature of the setting, the results are not really comparable with ours.

In our study, about 64% and 34% of the interventions were related to the appropriateness of therapy, including adding or discontinuing medication, and adjustment of the dosing regimen, respectively. These interventions yielded a cost avoidance of QAR 522,603 (USD 143,179), whereby the need for dose adjustment and cessation of medications was most prevalent among the anti-infective agents. Many anti-infective agents require renal dosage adjustment [30]. The addition of cardiovascular medications as prophylactic agents was also very common. All hospitalised patients are at risk of developing venous thromboembolism (VTE) and, thus, VTE prophylaxis is prescribed as part of the initial assessment upon admission and in the follow-up daily rounds [31].

Evaluating the distribution of medication classes in our study showed that the majority of the interventions were pertaining to anti-infective agents and cardiovascular medications. Hence, special attention should be given to these special pharmacological classes. Also to note, around 15% of all the interventions took place among critically ill patients. Many critically ill patients receive antibiotics during their ICU stay, in addition to that the infectious disease burden is high in the ICU setting [32]. Previous studies also demonstrated that around 60% of antibiotics prescribed in ICUs are inappropriate, which is higher than in other clinical wards [33, 34].

Therapeutic drug monitoring (TDM), which is one of the fundamental responsibilities of pharmacists, contributed to a cost avoidance of QAR 22,257 (USD 6,098) [35]. Clinical pharmacists do not only have a major role in preventing inappropriate use of medicines, but they also contribute to preventing disease-related problems. Clinical pharmacists have a profound role in recommending interventions related to laboratory, diagnostic, and culture tests, which resulted in the added cost of QAR 3,487 (USD 955) and a cost avoidance of QAR 28,627 (USD 7,843).

Our findings are likely to assist decision and policy makers in better judging the need for clinical pharmacy services, including the justification of salaries for personnel. In addition, our work provides healthcare professionals with evidence in relation to the main contributing factors of the cost associated with MRPs among the diverse Qatari population and, therefore, provides a better differential understanding of the burden of these, beyond the clinical and humanistic aspects.

This study has some limitations that should be acknowledged. Generalizability-wise, the analysis only included the internal medicine ward, critical care, and emergency units, and did not include other units such as urology, surgery, and stroke. However, the study sites represent the most important and busiest clinical areas, especially in relation to the incidence of MRPs. Also, our study was a retrospective study design that is highly susceptible to information and selection bias. Also, estimating probabilities of preventable ADEs was experience-based by local healthcare experts, and this could include a component of subjectivity and evaluation bias. This is an inherent limitation with the Nesbit et al. method [17], however. An additional limitation is that the cost of ADEs was calculated under the assumption that it was equal to an additional two days of hospital stay, which may, in reality, vary based on the type of ADE. This is added to that the source of this assumption is a nephrology-unit based study. However, given that no ADEs actually took place after interventions, it was impossible to accurately calculate the cost of ADEs in this study. In addition, there are no available reports of the cost of ADEs in general hospital settings in Qatar. Here, the cost of days of hospital stay as an assumed ADE cost in this study is not based on an assumed direct relevance between a nephrology units and general hospital wards, but is based on the believe that this is a relative underestimation of the ADE cost, which will consequently underestimate the economic benefit of the clinical pharmacy interventions in this study. To emphasize, the assumption here is the 2 days of hospital stay and not their nephrology-unit monetary value. In other wards, the value of the 2 days of hospital stay was based on the wards as were involved in our study. Furthermore to the limitations, we only included direct medical costs, which may not represent the real overall economic benefits, including the social, especially if we are to consider extended follow-up durations after the intervention. Finally, for future studies to consider, this study did not incorporate a content auditing assessment of the interventions.

## Conclusions

The deployment of clinical pharmacists in general clinical wards represents a critical approach for the management of MRPs in patients, associated positive reduction in ADEs in patients. This consequently contributes to considerable positive economic benefits and is worth expanding in large hospitals. Taking into consideration our perspective and limitations, this is likely to lead to a total benefit of QAR 566,759 (USD 155,276), and a total annual benefit of QAR 2,267,036 (USD 621,106) because of ADEs prevention. Noting that the resource constraints are increasingly placed on healthcare systems, the operation of clinical pharmacy services in HGH seems to need to be maintained at least.

## Supporting information

S1 File Ethics approval letter. (PDF)

S1 Data Base-case raw data. (XLSX)

## References

[1] Figueras A. The use of drugs is not as rational as we believe… but it can’t be! The emotional roots of prescribing. European journal of clinical pharmacology. Germany; 2011. pp. 433–435. doi: 10.1007/s00228-011-1024-5 21431396

[2] Mamo DB, Alemu BK. Rational Drug-Use Evaluation Based on World Health Organization Core Drug-Use Indicators in a Tertiary Referral Hospital, Northeast Ethiopia: A Cross-Sectional Study. Drug Healthc Patient Saf. 2020;12: 15–21. doi: 10.2147/DHPS.S237021 32021478 PMC6970620

[3] Medication Errors and Adverse Drug Events. In: September 7, 2019 [Internet]. 2019. https://psnet.ahrq.gov/primer/medication-errors-and-adverse-drug-events#:~:text=Each year%2C ADEs account for, common types of inpatient errors.

[4] Jourdan J-P, Muzard A, Goyer I, Ollivier Y, Oulkhouir Y, Henri P, et al. Impact of pharmacist interventions on clinical outcome and cost avoidance in a university teaching hospital. Int J Clin Pharm. 2018;40: 1474–1481. doi: 10.1007/s11096-018-0733-6 30367375

[5] Bond CA, Raehl CL. Adverse drug reactions in United States hospitals. Pharmacotherapy. 2006;26: 601–608. doi: 10.1592/phco.26.5.601 16637789

[6] Tariq RA, Vashisht R, Sinha A, Scherbak Y. Medication Dispensing Errors And Prevention. Treasure Island (FL); 2021. 30085607

[7] Lee M-S, Lee J-Y, Kang M-G, Jung J-W, Park H-K, Park H-K, et al. Cost implications of adverse drug event-related emergency department visits—a multicenter study in South Korea. Expert Rev Pharmacoecon Outcomes Res. 2020;20: 139–146. doi: 10.1080/14737167.2019.1608825 31012333

[8] Ruder AD, Smith DL, Madsen MT, Kass FH 3rd. Is there a benefit to having a clinical oncology pharmacist on staff at a community oncology clinic? J Oncol Pharm Pract Off Publ Int Soc Oncol Pharm Pract. 2011;17: 425–432. doi: 10.1177/1078155210389216 21248174

[9] ASHP guidelines: minimum standard for pharmacies in hospitals. American Society of Health-System Pharmacists. Am J Heal Pharm AJHP Off J Am Soc Heal Pharm. 1995;52: 2711–2717. doi: 10.1093/ajhp/52.23.2711 8601270

[10] Alzahrani AA, Alwhaibi MM, Asiri YA, Kamal KM, Alhawassi TM. Description of pharmacists’ reported interventions to prevent prescribing errors among in hospital inpatients: a cross sectional retrospective study. BMC Health Serv Res. 2021;21: 432. doi: 10.1186/s12913-021-06418-z 33957900 PMC8101218

[11] Dean B, Schachter M, Vincent C, Barber N. Causes of prescribing errors in hospital inpatients: a prospective study. Lancet (London, England). 2002;359: 1373–1378. doi: 10.1016/S0140-6736(02)08350-2 11978334

[12] Lewis PJ, Dornan T, Taylor D, Tully MP, Wass V, Ashcroft DM. Prevalence, incidence and nature of prescribing errors in hospital inpatients: a systematic review. Drug Saf. 2009;32: 379–389. doi: 10.2165/00002018-200932050-00002 19419233

[13] Franklin BD, McLeod M, Barber N. Comment on “prevalence, incidence and nature of prescribing errors in hospital inpatients: a systematic review”. Drug safety. New Zealand; 2010. pp. 163–166. doi: 10.2165/11319080-000000000-00000 20095075

[14] Kaboli PJ, Hoth AB, McClimon BJ, Schnipper JL. Clinical pharmacists and inpatient medical care: a systematic review. Arch Intern Med. 2006;166: 955–964. doi: 10.1001/archinte.166.9.955 16682568

[15] Hamad Medical Corporation. 2021. https://www.hamad.qa/EN/Pages/default.aspx

[16] Dooley MJ, Allen KM, Doecke CJ, Galbraith KJ, Taylor GR, Bright J, et al. A prospective multicentre study of pharmacist initiated changes to drug therapy and patient management in acute care government funded hospitals. Br J Clin Pharmacol. 2004;57: 513–521. doi: 10.1046/j.1365-2125.2003.02029.x 15025751 PMC1884463

[17] Nesbit TW, Shermock KM, Bobek MB, Capozzi DL, Flores PA, Leonard MC, et al. Implementation and pharmacoeconomic analysis of a clinical staff pharmacist practice model. Am J Heal Pharm AJHP Off J Am Soc Heal Pharm. 2001;58: 784–790. doi: 10.1093/ajhp/58.9.784 11351918

[18] Abushanab D, Gulied A, Hamad A, Abu-Tineh M, Abdul Rouf P V, Al Hail M, et al. Cost savings and cost avoidance with the inpatient clinical pharmacist interventions in a tertiary cancer care hospital. J Oncol Pharm Pract Off Publ Int Soc Oncol Pharm Pract. 2023; 10781552231160276. doi: 10.1177/10781552231160275 36946146

[19] Chen C-C, Hsiao F-Y, Shen L-J, Wu C-C. The cost-saving effect and prevention of medication errors by clinical pharmacist intervention in a nephrology unit. Medicine (Baltimore). 2017;96: e7883. doi: 10.1097/MD.0000000000007883 28834903 PMC5572025

[20] Qatar: Inflation rate from 1986 to 2026*. In: 2022 [Internet]. [cited 6 Jan 2022]. https://www.statista.com/statistics/379995/inflation-rate-in-qatar/

[21] Al MJ, van Hout BA, Michel BC, Rutten FF. Sample size calculation in economic evaluations. Health Econ. 1998;7: 327–335. doi: 10.1002/(sici)1099-1050(199806)7:4&lt;327::aid-hec342&gt;3.0.co;2-u 9683093

[22] Miyamoto GC, Ben ÂJ, Bosmans JE, van Tulder MW, Lin C-WC, Cabral CMN, et al. Interpretation of trial-based economic evaluations of musculoskeletal physical therapy interventions. Brazilian J Phys Ther. 2021;25: 514–529. doi: 10.1016/j.bjpt.2021.06.011 34340933 PMC8536890

[23] Allard J, Carthey J, Cope J, Pitt M, Woodward S. Medication errors: causes, prevention and reduction. Br J Haematol. 2002;116: 255–265. doi: 10.1046/j.1365-2141.2002.03272.x 11841425

[24] Bond CA, Raehl CL, Franke T. Clinical pharmacy services, pharmacist staffing, and drug costs in United States hospitals. Pharmacotherapy. 1999;19: 1354–1362. doi: 10.1592/phco.19.18.1354.30893 10600083

[25] van den Bemt PMLA, Postma MJ, van Roon EN, Chow M-CC, Fijn R, Brouwers JRBJ. Cost-benefit analysis of the detection of prescribing errors by hospital pharmacy staff. Drug Saf. 2002;25: 135–143. doi: 10.2165/00002018-200225020-00006 11888354

[26] 38- Qatar population (2021) live—Countrymeters [Internet]. Countrymeters.info. 2021 [cited 14 September 2021]. https://countrymeters.info/en/Qatar.

[27] Leape LL, Cullen DJ, Clapp MD, Burdick E, Demonaco HJ, Erickson JI, et al. Pharmacist participation on physician rounds and adverse drug events in the intensive care unit. JAMA. 1999;282: 267–270. doi: 10.1001/jama.282.3.267 10422996

[28] Al-Somai N, Al-Muhur M, Quteimat O, Hamzah N. The impact of clinical pharmacist and ID intervention in rationalization of antimicrobial use. Saudi Pharm J SPJ Off Publ Saudi Pharm Soc. 2014;22: 516–521. doi: 10.1016/j.jsps.2014.02.003 25561863 PMC4281637

[29] de Cazarim MS, Rodrigues JPV, Calcini PS, Einarson TR, Pereira LRL. Cost-benefit analysis of pharmacist interventions over 36 months in a university hospital. Rev Saude Publica. 2020;54: 94. doi: 10.11606/s1518-8787.2020054001895 33027344 PMC7524206

[30] Roughead EE, Semple SJ, Rosenfeld E. The extent of medication errors and adverse drug reactions throughout the patient journey in acute care in Australia. Int J Evid Based Healthc. 2016;14: 113–122. doi: 10.1097/XEB.0000000000000075 26886682

[31] Skeik N, Westergard E. Recommendations for VTE Prophylaxis in Medically Ill Patients. Ann Vasc Dis. 2020;13: 38–44. doi: 10.3400/avd.ra.19-00115 32273920 PMC7140153

[32] Brusselaers N, Vogelaers D, Blot S. The rising problem of antimicrobial resistance in the intensive care unit. Ann Intensive Care. 2011;1: 47. doi: 10.1186/2110-5820-1-47 22112929 PMC3231873

[33] Luyt C-E, Bréchot N, Trouillet J-L, Chastre J. Antibiotic stewardship in the intensive care unit. Crit Care. 2014;18: 480. doi: 10.1186/s13054-014-0480-6 25405992 PMC4281952

[34] Al-Maliky GR, Al-Ward MM, Taqi A, Balkhair A, Al-Zakwani I. Evaluation of antibiotic prescribing for adult inpatients at Sultan Qaboos University Hospital, Sultanate of Oman. Eur J Hosp Pharm Sci Pract. 2018;25: 195–199. doi: 10.1136/ejhpharm-2016-001146 31157018 PMC6452412

[35] Ratanajamit C, Kaewpibal P, Setthawacharavanich S, Faroongsarng D. Effect of pharmacist participation in the health care team on therapeutic drug monitoring utilization for antiepileptic drugs. J Med Assoc Thai. 2009;92: 1500–1507. 19938743

