# Supplementary material for: Economic impact of clinical pharmacist interventions in a general tertiary hospital in Qatar
Source: PLoS One. 2023 Jun 1;18(6):e0286419. doi: 10.1371/journal.pone.0286419 (PMC10234553; doi:10.1371/journal.pone.0286419)
Supplement: S1 File — (PDF) [file pone.0286419.s001.pdf]

مؤسسة حمد الطبية
Hamad Medical Corporation
HEALTH • EDUCATION • RESEARCH صحة • تعليم • بحوث

APPROVAL LETTER
MEDICAL RESEARCH CENTER
HMC, DOHA-QATAR

**Ms. Dina H. F. Abushanab**<br>**Staff Pharmacist**<br>**Department of Pharmacy**<br>**Women's Wellness and Research Center**	**Date: 18 September 2019**
**Protocol No.**	MRC-01-19-110
**Study Title:**	The economic impact of clinical pharmacists' interventions at Hamad Medical Corporation in Qatar.
The above titled research study has been approved to be conducted in HMC and is summarized below:	
**Study Type:**	Data Review
**Data Collection Period:**	01 March 2018 - 31 January 2019
**Hospitals/ Facilities Approved:**	Al Wakra Hospital (AWH),Communicable Disease Center (CDC),Hamad General Hospital (HGH),Heart Hospital (HH),National Center for Cancer Research (NCCCR),Women's Wellness and Research Center
**Team member list:**	Dr. Anas Ahmad E A Hamad , Dr. Daoud Al-Badriyeh , Dr. Maguy Saffouh El Hajj , Dr. Moza Sulaiman H Al Hail , Dr. Mohammad Issam Diab , Mr. Binny Thomas , Mr. Palli Valappila Abdul Rouf , Mr. Wessam Mohammed Elkassem , Ms. Dina H. F. Abushanab , Ms. Rasha Zakariya El Enany
**Review Type:**	\`Exempt\` under MOPH guidelines \`\`Category 3: Research involving the collection or study of existing: Data, documents, records and the information is recorded by the investigator in such a manner that subjects cannot be identified, directly or through identifiers linked to the subjects\`\`.
**Decision:**	Approved

This study must be conducted in full compliance with all the relevant sections of the Rules and Regulations for Research at HMC and the Medical Research Center should be notified immediately of any proposed changes to the study protocol that may affect the \`exempt\` status of this study. Wherever amendments to the initial protocol are deemed necessary, it is the responsibility of the Principal Investigator to ensure that appropriate reviews and renewed approvals are in place before the study will be allowed to proceed.

Please note that only official, stamped versions of the approved documentation are to be utilized at any stage in the conduct of this study. The research team must ensure that progress on the study is appropriately recorded in ABHATH, the online research system of the Medical Research Center.

We wish you success in this research and await the outcomes in due course.

Thank you

Ms. Emma Louise Pendleton
Assistant Director Business Development & Research
Medical Research Center- HMC

Medical Research Centre · Hamad Medical Corporation - Doha - Qatar · حمد Hamad

Date:18 September 2019
